# The innate and adaptive infiltrating immune systems as targets for breast cancer immunotherapy

**DOI:** 10.1530/ERC-16-0404

**Published:** 2017-02-13

**Authors:** Andrew M K Law, Elgene Lim, Christopher J Ormandy, David Gallego-Ortega

**Affiliations:** 1 Tumour Development GroupThe Kinghorn Cancer Centre, Garvan Institute of Medical Research, Darlinghurst, New South Wales, Australia; 2 Cancer Biology LaboratoryThe Kinghorn Cancer Centre, Garvan Institute of Medical Research, Darlinghurst, New South Wales, Australia; 3 Connie Johnson Breast Cancer Research LaboratoryThe Kinghorn Cancer Centre, Garvan Institute of Medical Research, Darlinghurst, New South Wales, Australia; 4 St. Vincent’s Clinical SchoolFaculty of Medicine, University of New South Wales Australia, Sydney, New South Wales, Australia

**Keywords:** breast cancer, MDSCs, tumour-infiltrating immune cells, immunotherapy

## Abstract

A cancer cell-centric view has long dominated the field of cancer biology. Research efforts have focussed on aberrant cancer cell signalling pathways and on changes to cancer cell DNA. Mounting evidence demonstrates that many cancer-associated cell types within the tumour stroma co-evolve and support tumour growth and development, greatly modifying cancer cell behaviour, facilitating invasion and metastasis and controlling dormancy and sensitivity to drug therapy. Thus, these stromal cells represent potential targets for cancer therapy. Among these cell types, immune cells have emerged as a promising target for therapy. The adaptive and the innate immune system play an important role in normal mammary development and breast cancer. The number of infiltrating adaptive immune system cells with tumour-rejecting capacity, primarily, T lymphocytes, is lower in breast cancer compared with other cancer types, but infiltration occurs in a large proportion of cases. There is strong evidence demonstrating the importance of the immunosuppressive role of the innate immune system during breast cancer progression. A consideration of components of both the innate and the adaptive immune system is essential for the design and development of immunotherapies in breast cancer. In this review, we focus on the importance of immunosuppressive myeloid-derived suppressor cells (MDSCs) as potential targets for breast cancer therapy.

## Introduction

In 1863 Rudolf Virchow, ‘the father of modern cellular pathology’, hypothesised a link between microinflammation and subsequent cancer development (Balkwill & Mantovani 2001). The concept of harnessing the power of the immune system to control cancer was later postulated by Paul Ehrlich in 1909 (Dunn *et al*. 2002). However, the mechanism underlying this only became better understood when the cellular components of innate and adaptive immunity, along with the molecular mechanisms that cancer cells utilise to subvert and hide from the immune system, began to be uncovered (Burnet 1957*a*,*b*). Our knowledge of the interface of cancer and immune cells in tumours is growing exponentially, revealing new molecular pathways and opening therapeutic interventions. The use of monoclonal antibodies against neoantigens; the development of cancer vaccines; adoptive transfer of *in vitro*-engineered cancer-reactive lymphocytes; immunomodulatory agents such as cytokines or toll-like receptor agonists and more recently, checkpoint inhibitor blockade are some of the therapeutic approaches with proven success in several types of cancer.

The low immunogenicity and intense immunosuppressive environment of breast tumours limit the benefit of immunotherapies targeting the adaptive immune system, such as checkpoint inhibitors (Rugo *et al*. 2015). Mechanisms of immunosuppression are essential for the normal functioning of the mammary gland during development (Clarkson *et al*. 2004, Stein *et al*. 2004, 2009). These same mechanisms might be hijacked by breast cancer cells to promote tumour tolerance and escape immune surveillance at the early stages of tumour formation, underscoring the importance of the innate immune system during breast cancer progression.

**Figure 1:**
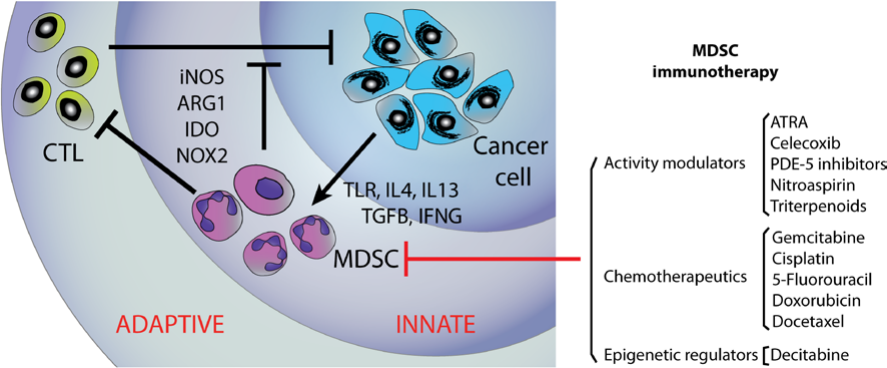
Schematic representation of the two interconnected layers of the tumour-infiltrating immune system, illustrating the molecular pathways of induced tumour tolerance driven by MDSCs. A summary of potential therapeutic approaches to target MDSCs are represented on the right hand side.

In this review, we discuss the importance of the immune system during normal mammary gland development and the therapeutic strategies to target the two inter-related immune layers that operate in tumours; the adaptive immunity that directly exerts cancer-rejecting functions represented by cytotoxic T lymphocytes (CTLs) and the regulatory innate layer that orchestrates immune suppression, represented by myeloid-derived suppressor cells (MDSCs) (Fig. 1). Emerging evidence indicates that MDSC is key for all stages of the carcinogenic process. They are recruited by tumour-derived factors and are highly influential on other cell populations in the tumour microenvironment, for example, displaying a number of mechanisms able to suppress T-cell cytotoxic activity. A number of existing therapeutics have serendipitous activity against MDSC. The development of new strategies to overcome immune suppression is imperative to improve immunotherapy and the therapeutic potential of defining and specifically targeting the active MDSC population has attracted increasing interest in recent years.

## The role of the immune system in cancer

There is mounting evidence demonstrating a central role of the immune system in cancer. Immune cell populations co-evolve with cancer cells and sculpt the progression of the tumour, producing sustained inflammatory pathways that suppress immune rejection, thus cooperating with cancer cells to promote tumour growth and spread, including the preparation of the premetastatic niche (Balkwill & Mantovani 2001, Dunn *et al*. 2002, Smyth *et al*. 2006, Sceneay *et al*. 2012). In the early stages of carcinogenesis, tumour cells are rejected by an innate immune mechanism also referred to as immunosurveillance (Dunn *et al*. 2002). This process is mainly driven by natural killer (NK) cells that are activated and recruited in response to tissue damage signals, mainly interferon gamma (IFNG) originating from neighbouring cells that are activated by the abnormal growth of cancer cells (Dunn *et al*. 2002). These cells control the otherwise frequent appearance of neoplasms. Cancer immunoediting is the process by which the immune system protects the host from tumour development, offering a selection pressure for the fittest and yet non-immunogenic cancer clones that will finally populate and generate the clinically detectable tumour (Dunn *et al*. 2002). Thus, immunoediting implies that clinically relevant tumours have developed mechanisms to evade immune surveillance and induce tumour tolerance. These mechanisms include decreased expression of the major histocompatibility complex (MHC) I and other co-stimulatory molecules and expression of immunosuppressive factors that contribute to escape from immune recognition. The resulting tumours display a strong immune suppressive tumour microenvironment and fail to elicit an appropriate adaptive immune response, with multiple molecular mechanisms in place to interfere with CTLs, resulting in poor infiltration of reactive tumour-rejecting T-cells. The CTLs that do infiltrate present symptoms of immune exhaustion (Dushyanthen *et al*. 2015). The current understanding of the dichotomous nature of immune cells in tumours is that IFN-γ-producing CD4+ T helper (Th) 1 and CD8+ T lymphocytes, along with mature dendritic cells (DCs), NK cells, M1 macrophages, and type 1 NK T-cells can generate anti-tumour responses; and conversely, CD4+ Th2 cells, CD4+ T regulatory (Treg) and type 2 NK T-cells, MDSC, immature DCs, or alternatively activated (M2) macrophages promote tumour tolerance and support tumour growth and progression (Gobert *et al*. 2009, Ruffell *et al*. 2010, Zamarron & Chen 2011, Emens 2012).

### Immunity associated with mammary development

The mammary gland is a unique organ because it develops post-natally, undergoing a profound morphogenesis during puberty and with each round of pregnancy (Oakes *et al*. 2014). These processes are tightly controlled by hormones. Oestrogens, progesterone and prolactin act on the mammary epithelium in synergy with corticosteroids and growth hormone to orchestrate mammary gland development (Brisken & O’Malley 2010, Macias & Hinck 2012). These events encompass epithelial cell differentiation and stromal interactions (Robinson *et al*. 1999, Arendt *et al*. 2010), including the generation of new blood vessels, infiltration of immune and inflammatory cells, fibroblast reorganisation and the loss and gain of lipid droplets within adipocytes (McCready *et al*. 2010).

Immune cells play an essential role in mammary gland remodelling during normal development. Macrophages and eosinophils are key players during ductal outgrowth and branching morphogenesis, in a process controlled by CSF1 (Gouon-Evans *et al*. 2000, Coussens & Pollard 2011). Antibody-presenting cells (APCs) associated with mammary epithelial cells, presumably macrophages, communicate with CD4+ Th1 cells to suppress ductal development and branching. In this mechanism, CD4+ Th1-type cell-secreted IFNG directly inhibits luminal epithelial differentiation (Plaks *et al*. 2015).

During involution, the process by which the mammary gland returns to its non-lactating state, the immune system plays a particularly important role. Milk stasis and mammary engorgement after weaning results in a wave of apoptosis in mammary epithelial cells (Macias & Hinck 2012). Apoptosis in early involution is induced by local factors, decreased expression of milk proteins and block of prolactin-mediated survival signal transduction (Li *et al*. 1997). During this stage, cell death occurs despite high levels of systemic lactogenic survival factors like prolactin (PRL), glucocorticoids (GCs) and insulin-like growth factors (IGF-I) (Feng *et al*. 1995, Hadsell & Abdel-Fattah 2001, Oakes *et al*. 2008*b*). As involution progresses, systemic pro-survival action of PRL and IGF-I is inhibited by local factors such as TGFB and IGFBP5; and together with systemic lower levels of PRL and GC contribute to establish irreversible involution (Feng *et al*. 1995, Hadsell & Abdel-Fattah 2001, Tonner *et al*. 2002).

Gene expression profiling studies have identified an involution-associated immune response that is similar to a wound-healing process (Stein *et al*. 2004, 2009). Apoptosis of the alveolar cells during involution triggers an immune cascade that co-ordinately recruits the immune system. This immune response is carefully orchestrated to avoid a robust inflammatory reaction, ensuring a safe clearance of cellular debris and residual milk. The first immune cells recruited to the involuting gland are phagocytic neutrophils, presumably recruited by CXCL1 signalling and accompanied by the expression of pro-inflammatory mediators (IL1a, IL1b and IL13). At this stage, transcriptional evidence of immunosuppressive cytokines is also detected, presumably released by viable epithelial cells, indicating an exquisite control of inflammation (Clarkson *et al*. 2004, Stein *et al*. 2004), resulting in the prevention of neutrophil extravasation (Clarkson *et al*. 2004). Subsequently, macrophages and eosinophils are recruited once involution becomes irreversible through CXCL14 signalling (Monks *et al*. 2002, Stein *et al*. 2004). A strong innate immune signature, characterised by acute phase response (APR) genes, is detected during involution (Clarkson *et al*. 2004, Stein *et al*. 2004). The APR aims to minimise tissue damage by suppressing inflammation, reinforcing the idea that involution is a highly controlled inflammatory event. The main function of these cells is to remove dying cells by phagocytosis, but the immune cells are also a source of MMPs required for the matrix remodelling (Watson & Kreuzaler 2011). Finally, recruitment of plasma B cells is found late in involution and is characterised by the upregulation of a number of immunoglobulin genes. Although the role of these B cells is still controversial, they are not part of an adaptive immune response, as phagocytic macrophages of involuting mammary glands are not capable of antigen presentation to T-cells (Monks *et al*. 2002). The involuting microenvironment has been suggested to promote cancer development, and it is associated with transiently enhanced risk of breast cancer (McDaniel *et al*. 2006, Polyak 2006, Schedin 2006, Schedin *et al*. 2007). The wound-healing involution signature is associated with metastatic breast cancer (Stein *et al*. 2009).

### Breast cancer and its immunogenicity

Breast cancer is a very heterogeneous disease (Polyak 2011), both histologically (Lakhani *et al*. 2012) and molecularly (Cancer Genome Atlas Network 2012), with transcriptional profiles defining at least 5 intrinsic molecular subtypes (Perou *et al*. 2000, Prat *et al*. 2010) that correlate with clinical outcome (Sorlie *et al*. 2001). Breast cancer has been traditionally classified based on the presence of the receptors for the steroid hormones oestrogen (ER) and progesterone (PR), and the epidermal growth factor receptor family member HER2. ER and PR are part of the nuclear receptor superfamily of ligand-regulated transcription factors essential for the self-renewal and replicative potential of the mammary gland (Brisken & O’Malley 2010). In breast cancer, ER and PR are not only markers for diagnosis but their signalling plays a major role in disease progression, reviewed in Carroll *et al*. (2016). Approximately 70% of breast cancers express ER and are considered to be ER driven, as such, oestrogen deprivation is the standard-of-care treatment for ER+ breast cancer; however, resistance to endocrine therapy remains one of the most important clinical challenges as it frequently results in metastatic lethal breast cancer, reviewed in Ma *et al*. (2015) and Musgrove and Sutherland (2009). PR signalling is increasingly attracting the attention for the treatment of breast cancer as several strategies to target this pathway are undergoing at different stages of clinical development, including the next generation of selective progesterone receptor modulators (SPRMs) and RANKL (denosumab) and WNT inhibitors (Anastas *et al*. 2012, Brisken 2013).

The catalogue of somatic mutations found in each tumour type is indicative of the likelihood of the formation of antigens that differentiate cancer cells from their non-transformed counterparts (Schumacher & Schreiber 2015). These neoantigens are often products of mutated cellular genes, aberrantly expressed normal genes or genes-encoding viral proteins. The prevalence of somatic mutations in breast tumours is comparable to many other tumours of solid origin (ranging 33–66 per tumour) but much lower compared to the highly immunogenic and highly mutated tumours such as melanoma or lung cancer that display about 200 non-synonymous mutations per tumour (Alexandrov *et al*. 2013, Vogelstein *et al*. 2013). The mutation rate is lowest in luminal A molecular subtype and highest in the basal-like and HER2 subtypes (Cancer Genome Atlas Network 2012). Breast cancers have low expression of MHC antigens and co-stimulators. The breast, due to its dramatic tissue remodelling with changes in reproductive state, also has a natural immunosuppressive–permissive microenvironment, and together, these features create the relatively low state of immunogenicity of breast cancer. In support of this, the incidence of breast cancer is not significantly higher in therapeutically immunosuppressed populations (Penn 1988, Gallagher *et al*. 2010).

Despite the fact that cancer-associated immunogens are not highly common in breast cancer, the literature provides clear examples of neoantigen recognition and the generation of an immune response. In a small patient cohort with HER2+ breast cancer, Disis and coworkers demonstrated CD4+ helper/inducer T-cell immunity and antibody-mediated immunity to HER-2/neu protein (Disis *et al*. 1994). A lower level of HER2 T-cell immunity has been proposed as a prognostic marker of increased risk of treatment failure in invasive breast carcinoma patients (Datta *et al*. 2016).

The intensity of the tumour–immune interaction varies in each breast cancer subtype. Gene expression analysis has identified breast tumours that present with high levels of immunomodulatory gene activation (Rody *et al*. 2009, Yau *et al*. 2010). These signatures are prognostic, particularly in the triple-negative (ER−, PR− and Her2−) and HER2+/ER− breast cancer subtypes (Desmedt *et al*. 2008, Rody *et al*. 2009, Yau *et al*. 2010). In luminal breast cancer, a high B cell/plasma cell signature was found to be prognostic in patients with more highly proliferative ER+ breast cancer who received tamoxifen treatment, but had no prognostic value in patients with low-proliferative ER+ cancer (Bianchini *et al*. 2010). In general, patients having tumours with a Th1 CTL cytokine profile have a better prognosis than those with a Th2 profile or a pattern of tumour-associated macrophages (TAM) infiltration via CSF1 recruitment (DeNardo *et al*. 2011).

The infiltrating immune component of breast tumours has been used to as a prognostic and predictive biomarker to chemotherapy and radiotherapy (Apetoh *et al*. 2007, Ladoire *et al*. 2011, Ruffell *et al*. 2012, Loi *et al*. 2013*a*). One of the best-characterised immune-related prognostic factors is tumour lymphocytic infiltration (TILs) (Aaltomaa *et al*. 1992, Demaria *et al*. 2001, DeNardo & Coussens 2007, Schmidt *et al*. 2008, Denkert *et al*. 2010, Loi *et al*. 2013*b*, Maenhout *et al*. 2014). The infiltration of CD8+ T-cells is associated with better prognosis in ER– and ER+/HER2+ tumours, but no association was found in ER+ tumours (Baker *et al*. 2011, Mahmoud *et al*. 2011, West *et al*. 2011, Liu *et al*. 2012, Seo *et al*. 2013, Ali *et al*. 2014, Chen *et al*. 2014, Gallego-Ortega *et al*. 2015). The Treg marker FOXP3 was shown to predict worse survival (Criscitiello *et al*. 2014*a*) and to associate with distal metastasis-free survival; however, the clinical relevance of Tregs has been controversial, offering mixed results (Mahmoud *et al*. 2011, West *et al*. 2013, Ali *et al*. 2014). Patients with higher TIL infiltration in their tumours had an improved response to neoadjuvant chemotherapy (Denkert *et al*. 2010, Issa-Nummer *et al*. 2013) and improved survival (Loi *et al*. 2013*b*).

## Immunotherapy strategies in breast cancer: targeting the adaptive immune system

In ER+ breast cancer, immunomodulators with the potential to delay tumour recurrence after standard adjuvant endocrine therapy would have great benefit. The acquisition of therapy resistance is a common clinical challenge. Approximately 30% of patients with early-stage ER+ breast cancer treated with adjuvant endocrine therapy develop *de novo* anti-oestrogen therapy resistance (Musgrove & Sutherland 2009). Targeted therapies have recently been used in combination with ER-directed therapies to improve survival outcomes in patients with metastatic breast cancer. These include inhibitors of PI3K cell signalling pathway, such as Everolimus, an inhibitor of mTOR, which is downstream of PI3K (Bachelot *et al*. 2012, Baselga *et al*. 2012), and inhibitors CDK 4/6, which regulate cell cycle progression. Co-administration of palbociclib, a CDK 4/6 inhibitor, with ER-directed therapies approximately doubles progression-free survival compared with ER-directed therapy alone (Finn *et al*. 2015,^ 2016^, Turner *et al*. 2015). These combinations are associated with greater toxicities compared to endocrine therapy alone. The checkpoint inhibitor anti-CTLA4 in combination with the aromatase inhibitor exemestane was beneficial in the metastatic ER+ setting (Vonderheide *et al*. 2010). However, further characterisation of the infiltrating immune cells is necessary to determine their nature and functional role as well as the understanding of the differences in immunogenicity that exists in different subtypes of breast cancer (Criscitiello *et al*. 2014*b*, Loi *et al*. 2013*b*, Salgado *et al*. 2015). Stratification according to these parameters is key to fully address whether the combination with immunotherapy will produce more durable clinical responses and prevention of the acquisition of resistance in ER+ patients treated with endocrine therapy. An advantage of immunotherapy is that the immune system is able to target multiple antigens at the same time, rendering less likely the development of therapy resistance. Furthermore, once immune rejection is activated, vaccines may boost immune surveillance for residual disease without added toxicity. This approach is currently being explored in HER2 vaccine-based clinical trials (Mittendorf *et al*. 2014). Another potential advantage of immunotherapies is that their immediate effects can be used as surrogate makers to evaluate the efficacy of therapeutic intervention, which is of particular importance in neoadjuvant studies.

In patients with breast cancer, TILs have been shown to be associated with anti-oestrogen therapy resistance; in a neoadjuvant setting of ER+ postmenopausal women with early-stage ER+ breast cancer (I–IIIB), a poor aromatase inhibitor response was strongly associated with the expression of inflammatory response-related pathways and lymphocytic infiltration (Miller *et al*. 2009, Dunbier *et al*. 2013). This lymphocytic infiltration is presumably mediated by myeloid-driven activation of Tregs, as other reports characterising T-cell subtype found that T-cell infiltration is a good prognosis factor in breast cancer, in particular in ER– subtypes. This opens the question whether the behaviour of the immune system is differential in ER+ and ER− breast cancer and stresses the importance of the characterisation of the specific subset of lymphocytic infiltration as a predictive tool. Better preclinical models for the study of the association of infiltrated immune system and endocrine resistance are necessary, as cell lines and xenografts in immunodeficient mice are unable to model the contributions of TILs to therapy resistance (Chan *et al*. 2002, Martin *et al*. 2003, Lupien *et al*. 2010, Miller *et al*. 2010, Gee *et al*. 2011). Nonetheless, the combination of endocrine therapy with immunomodulators represents a potential avenue for the treatment of ER+ breast cancer.

### Therapy based on tumour-associated antigens

Immunotherapy with monoclonal antibodies targeting the HER2 protein, such as trastuzumab, have become the mainstream therapy for patients with HER2+ early- and late-stage breast cancer (Nuti *et al*. 2011). The immune basis of the therapeutic benefit has not been well understood compared to the effect of these therapies on the HER2 signalling pathway. The immune effects include: activation of both innate and adaptive immune systems, activating antibody-dependent cytotoxic cellular (ADCC) killing of HER2-overexpressing cells via NK cells (Clynes *et al*. 2000, Arnould *et al*. 2006, Barok *et al*. 2007); enhanced tumour surveillance by increasing INFγ production by NK cells, a process that is stimulated by IL-12 (Jaime-Ramirez *et al*. 2011) and eliciting an adaptive immune response based on HER2 presentation by HLA class I molecules to activate the anti-tumour activity of CD8+ T-cells and reduce Tregs (Perez *et al*. 2007, Horlock *et al*. 2009). Lapatinib, a dual tyrosine kinase inhibitor targeting downstream activation of EGFR1/HER1 and HER2, also engages the immune system by stabilising HER2 in the cell membrane, potentiating NK cell recognition of trastuzumab-bound HER2 (Mimura *et al*. 2011).

The use of therapeutic vaccines based on monoclonal antibodies directed against tumour-associated antigens to elicit a CTL response and tumour rejection is still under development in breast cancer. An example is E75 Nelipepimut-S, a human leukocyte antigen (HLA)-A2/A3-restricted immunogenic peptide derived from the HER2 protein (Mittendorf *et al*. 2014). Another example is hTERT-mediated immunity. hTERT activity is increased in >85% of all human cancers compared with that in normal cells (Kim *et al*. 1994). The hTERT catalytic subunit is recognised by cytotoxic T lymphocytes (Vonderheide *et al*. 1999). Importantly, naturally occurring CD8+ T-cells specific for the hTERT peptide I540 (ILAKFLHWL) have been observed in high numbers in blood from remission patients with different types of cancer (Gannagé *et al*. 2005, Filaci *et al*. 2006). Vaccination of patients with metastatic breast cancer with the I540 peptide in combination with GM-CSF resulted in increased TILs, which associated with necrotic areas and hTERT-specific immunity (Domchek *et al*. 2007). Another example is the human high-affinity folate-binding protein (FBP), which is a source of antigenic peptides recognised in ovarian cancer, which is also recognised in breast cancer. FBP is overexpressed in 50–70% of breast tumours and its epitopes are presented by HLA-A2 in these cancers. These peptides are efficient at amplifying the response of tumour-associated lymphocyte populations in terms of lytic function, enhanced proliferation and specific IFN-γ release (Peoples *et al*. 1999). Additional vaccine strategies are being investigated in patients with breast cancer such as MUC1 (Jerome *et al*. 1993, Brossart *et al*. 1999, Emens 2012), WT1 (Oka & Sugiyama 2010, Di Stasi *et al*. 2015) and NY-ESO-1 (Odunsi *et al*. 2014), including several ongoing or recently completed phase II studies. Other examples include HER2-derived peptide vaccines; an allogeneic GM-CSF1-secreting vaccine; a HER2 peptide-pulsed, dendritic cell vaccine; and PANVAC, which incorporates vaccinia and fowlpox viruses genetically engineered to express the tumour-associated antigens carcinoembryonic antigen and MUC1.

### Checkpoint inhibitors

Checkpoint inhibitors such as antibodies against CTLA4 and PD-1 have elicited remarkable responses against cancers with high numbers of neoantigens, such as melanoma, lung cancer and renal cell carcinoma (Hodi *et al*. 2010, Brahmer *et al*. 2012, Topalian *et al*. 2012, Herbst *et al*. 2014, Powles *et al*. 2014, Tumeh *et al*. 2014, Rizvi *et al*. 2015, Robert *et al*. 2015), but have also promoted responses in ‘less immunogenic’ solid tumours (Le *et al*. 2015, Ojalvo *et al*. 2015, Shah *et al*. 2015), including triple-negative breast cancer (TNBC) (Emens & Middleton 2015, Nanda *et al*. 2016). One of the main challenges of checkpoint inhibitor blockade therapy is that many patients have low levels of TILs, especially in ER+ luminal breast cancer, whereby the efficacy of checkpoint inhibitors has so far been disappointing (Rugo *et al*. 2015). The potential of T-cell-mediated therapy in breast cancer is likely to be achieved in combination with standard-of-care therapies, and there is evidence to suggest that the immune system is pivotal in determining the response to targeted therapy. Examples can be found in preclinical models of trastuzumab combined with CTLA4 treatment (Persson *et al*. 2011) or PD-L1 blockade (Park *et al*. 2010, Rakhra *et al*. 2010, Stagg *et al*. 2011). In a phase 1 clinical trial in patients with metastatic hormone-responsive breast cancer, Tremelimumab (anti-CTLA4) in combination with exemestane demonstrated an overall response rate (defined as stable disease for 12 weeks or more) in 11 of 26 patients (42%) (Vonderheide *et al*. 2010). Treatment was associated with increased levels of peripheral CD4+ and CD8+ T-cells that expressed the protein-inducible co-stimulator of T-cell activation (ICOS), a potential biomarker of immune activation resulting from blockade of CTLA4. A marked increase in the ratio of ICOS+ T-cells/FOXP3+ Tregs in the peripheral blood was observed (Tarhini *et al*. 2014). Other examples aimed at increasing tumour rejection given in combination with standard cancer treatments (Criscitiello *et al*. 2014*a*) include trastuzumab + anti-PD-1 therapy for HER2+ metastatic patients (PANACEA) and nelipepimut-S + trastuzumab (Mittendorf *et al*. 2015).

There is also evidence that standard non-targeted therapies elicit an immune response that is essential for complete patient response. Chemotherapy treatment with anthracyclines and platinum salts increases DC presentation of tumour antigens (Zitvogel *et al*. 2011), whereas taxanes are associated with an increase in lymphocyte infiltration in locally advanced breast cancer (Demaria *et al*. 2001) and increased Th-1-associated cytokines in metastatic breast cancer (Tsavaris *et al*. 2002). Finally, cyclophosphamide depletes Tregs (Zitvogel *et al*. 2011). Radiation therapy similarly elicits anti-tumour immune responses by boosting tumour antigen presentation and T-cell infiltration (Matsumura *et al*. 2008).

A critical concept underlying the current view of immunotherapy is that the ultimate end-effector and therapeutic target of cancer immunotherapy is the tumour-specific T-cell. An important limitation of these types of therapies is that they are based on the pre-existence of neoantigens that can be exploited as engineered therapeutic targets or in the reactivation of tumour rejection mechanisms in pre-existing T-cells. However, the majority of breast cancer tumours do not display elevated numbers of TILs, with the median percentage of stromal TILs reported as 10% in ER+ breast cancer, 15% in HER2+ breast cancer and 20% in TNBC, whereas the median intratumoral infiltration drops to 1.5, 3 and 5%, respectively (Loi *et al*. 2013*b*), thus limiting the therapeutic benefit of these approaches. Strategies to dismantle the strong immunosuppressive tumour microenvironment that precludes cytotoxic T-cell activity are also under investigation. The T-cell immunomodulatory enzyme indoleamine 2,3 dioxygenase (IDO) is a key pathway in causing T-cell dysfunction in cancer, facilitating immune escape (Prendergast 2008). In the immune-competent MMTV-*Neu* mouse model, small-molecule inhibitors of IDO potentiated the efficacy of cytotoxic drugs without increasing their side effects, demonstrating that immunotherapy and chemotherapy can be combined to more effectively destroy cancer cells (Muller *et al*. 2005). Although the mechanism of IDO and chemotherapy synergy is not clear, it has been suggested that cooperating cytotoxic agents may preferentially compromise the survival of regulatory T-cells, contributing to a weakening of immune tolerance and stimulation of anti-tumour immunity (Machiels *et al*. 2001, Mason *et al*. 2001, Nowak *et al*. 2003). Additionally, the aberrant angiogenesis in tumours creates an endothelial barrier that restricts the extravasation of tumour-rejecting T-cells and creates an immunosuppressive environment (Motz & Coukos 2011, Melero *et al*. 2014). TAM, MDSC and Treg cells are capable of both stimulating angiogenesis and supporting immunosuppression in the tumour microenvironment through the secretion of mediators such as VEGFA and PGE2 (Yang *et al*. 2004, Facciabene *et al*. 2011, Motz *et al*. 2014). Through these factors, T-cells accumulate predominantly in the stroma but are unable to properly infiltrate into the tumour, thus impeding the efficacy of immunotherapy (Buckanovich *et al*. 2008, Quezada *et al*. 2008). As such, strategies that combine immunotherapy with agents that eliminate mediators that induce both angiogenesis and immunosuppression are currently under investigations. For example, Basu and coworkers reported the administration of both a combination of celecoxib and a dendritic cell-based cancer vaccine was able to significantly reduce metastasis, tumour burden and increase survival in breast cancer (Basu *et al*. 2006). Anti-VEGF antibody has also been used to disrupt the tumour vasculature to increase the infiltration of T-cells from adoptive cell transfer (ACT)-based immunotherapies and has been found to greatly improve ACT-based immunotherapy (Shrimali *et al*. 2010).

A dominant immunosuppressive environment that selectively excludes T-cell tumour infiltration can be explained in the context of the innate immune system, driven by cancer-associated fibroblasts (CAFs), TAMs and MDSCs (Joyce & Fearon 2015). Using the poorly immunogenic and highly metastatic 4T1 mammary tumour model, Kim and coworkers (Kim *et al*. 2014) showed that elevated numbers of MDSCs were responsible for the failure of checkpoint inhibitor therapy. Targeting MDSCs with epigenetic modulators or Ly6G antibodies sensitised 4T1 tumours to checkpoint blockade resulting in effective combination therapy. These experiments underscore the importance of immunosuppressive innate immune cells as targets for breast cancer therapy.

## Immunotherapy strategies targeting the innate immune system in breast cancer: myeloid-derived suppressor cells

MDSCs are a heterogeneous population composed of precursors of the myeloid-cell lineage. They are found in inflammatory pathological conditions, such as infections and various types of cancer. Their recruitment to sites of inflammation is induced by pro-inflammatory cytokines and often they are localised abundantly in peripheral lymphoid organs and tumours, where they play an important role in immunosuppression of both the innate and adaptive immune system. Along with their immunosuppressive functions, MDSCs also stimulate tumour growth by promoting angiogenesis and tumour cell survival (Condamine *et al*. 2015) and facilitate local invasion and distant metastasis, preparing the premetastatic niche in distant tissues including lung, by inducing mesenchymal-to-epithelial transition (Erler *et al*. 2009, Yan *et al*. 2010, Gao *et al*. 2012, Sceneay *et al*. 2012), brain (Liu *et al*. 2013) and bone (Danilin *et al*. 2012, Sawant *et al*. 2013).

### Classification of MDSCs

MDSCs may be broadly classified into monocytic and polymorphonuclear granulocytic subtypes (M-MDSC and PMN-MDSC, respectively) based on different expression of cell surface markers. In mice, MDSCs express high levels of CD11b, also known as integrin α M, and Gr1, a granulocytic marker that is composed of the macrophage and neutrophil markers, Ly6C and Ly6G, respectively. The level of expression of these markers is frequently used to classify murine MDSC into the two subtypes (Ostrand-Rosenberg & Sinha 2009). M-MDSCs are mononuclear and have of high levels of Ly6C and low or absent Ly6G (CD11b^+^Ly6G^low/−^Ly6C^+^), whereas PMN-MDSC have multi-lobed nuclei and expresses Ly6G and low Ly6C (CD11b^+^Ly6G^+^Ly6C^low^). These two subtypes have been found to have different mechanisms of T-cell suppression and both are expanded in cancer (Movahedi *et al*. 2008).

In humans, a marker that is homologous to the murine Gr1 antigen is absent and instead identification is based on the myeloid-cell markers CD11b^+^, CD33^+^, HLA-DR^low/−^ and negative for lineage-specific antigen (Lin^−^). M-MSDC is typically CD11b^+^CD33^+^CD14^+^HLA-DR^−^ and PMN-MDSC are characterised by CD11b^+^CD33^+^CD15^+^HLA-DR^−^. The phenotypic markers used to identify MSDCs can vary based on the context of the disease, as distinct subtypes of MSDCs have been isolated from different cancers, and often, different studies utilise specific combinations of markers for a particular subsets of MDSCs (Filipazzi *et al*. 2007, Hoechst *et al*. 2008, Vetsika *et al*. 2014). As MDSCs are composed of a heterogenous population of immature myeloid cells (IMCs) at various stages of differentiation, it is to be expected that such disparity in the phenotypic markers of MDSCs exists. Thus, it is essential to identify markers that better define unique MDSC subpopulations to develop better therapies aimed at MDSC modulation.

### Developmental biology of MDSCs

Under normal conditions, IMCs are generated in the bone marrow and lack immunosuppressive activity. In acute pathological conditions, such as infections or trauma, IMC is released from the bone marrow, and the population of MDSCs is expanded through the differentiation of common myeloid progenitor cells. The immunosuppressive activity of MDSCs is then used to mediate and suppress the immune response to prevent any harm caused by an overstimulated immune system. Once the inflammation is resolved, myelopoiesis reverts to a steady basal level. A chronic inflammation setting, such as cancer, leads to a continuous MDSC expansion. Accumulation of MDSCs occurs in the bone marrow and in peripheral lymphoid organs and is induced in response to chronic exposure to growth factors secreted by tumours (Almand *et al*. 2001, Sica & Bronte 2007). Within the tumour microenvironment, many of these factors are pro-inflammatory cytokines, such as IL1, IL6, PGE2, S100 proteins, GM-CSF, M-CSF, VEGF and TNFA, which promote an aberrant state of myelopoiesis (Sawanobori *et al*. 2008, Ostrand-Rosenberg & Sinha 2009). Consequently, this increases the levels of MDSCs, and in both patients, with breast cancer and in mouse models, this effect is seen most prominently associated with high tumour burden (Marigo *et al*. 2008). The MDSCs then subsequently migrate into tumours, where they exert their immunosuppressive effects. As such, MDSCs play a major role in reducing the efficacy of immunotherapies and promoting resistance against treatments (Mantovani 2010, Gabrilovich *et al*. 2012).

### Recruitment and expansion of MDSCs

**Figure 2:**
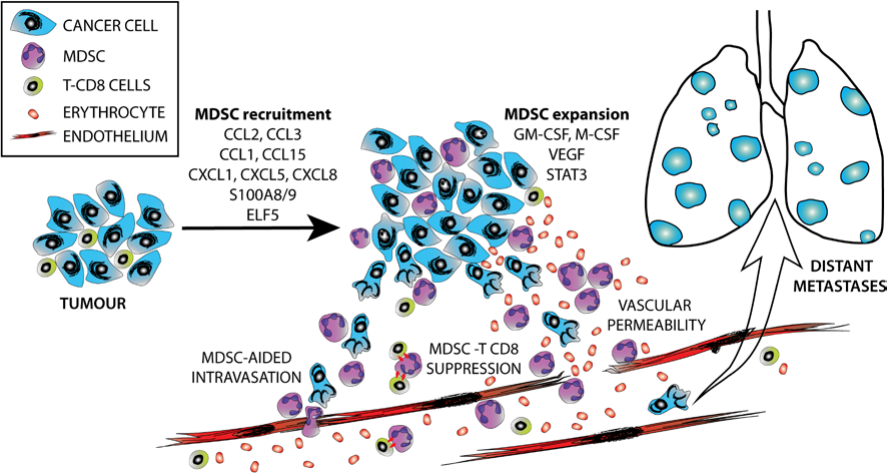
Schematic representation of the functions of infiltrating MDSCs during cancer progression and metastatic spread, depicting some known molecular mechanisms.

MDSCs are found abundantly in tumours in tumour-bearing mice and have been isolated from patient-derived cancers, including breast cancer (Yu *et al*. 2013, Templeton *et al*. 2014, Gentles *et al*. 2015). The recruitment of MDSCs to tumours is governed by the same factors and mechanisms that regulate the migration of neutrophils and monocytes. M-MDSC and monocytes are recruited to primary tumour sites and metastatic sites by chemokines secreted by tumour cells, most commonly CCL2 and CCL5, which function to regulate monocyte chemotaxis (Qian *et al*. 2011). These chemokines primarily recruit the M-MDSC subtype and are implicated in different cancers, such as breast cancer (Yu *et al*. 2013). Kitamaru and coworkers reported the recruitment of inflammatory monocytes to breast tumours via a CCL2-induced chemokine cascade. These were retained within the tumour by CCL3, a chemokine produced by metastasis-associated macrophages through the activation of the CCL2 receptor (CCR2) (Kitamura *et al*. 2015). Similarly, CCL2 and CCL3 recruit PMN-MDSC to tumours (Reichel *et al*. 2012, Chun *et al*. 2015) (Fig. 2). Furthermore, hypoxic conditions in the tumour microenvironment can further promote the recruitment and expansion of MDSCs, leading to a weakened antitumor response (Sceneay *et al*. 2012). Reactive oxygen species (ROS) produced by MDSCs can also lead to nitration of CCL2 (N-CCL2), which has been found to contribute to infiltrated T-cell exclusion, trapping T lymphocytes in the stroma that surrounds the tumour (Molon *et al*. 2011). Other factors and chemokines that have been found to recruit MDSCs include CCL1, CCL15 and CXC chemokines, such as CXCL1, CXCL5, CXCL8 and S100A8/9 proteins (Kumar *et al*. 2016*b*).

The molecular effectors involved in these MDSC recruitment mechanisms are poorly understood. A transcriptional network that operates in luminal breast cancer has been identified that promotes MDSC recruitment; this network is regulated by the ETS transcription factor ELF5 (Gallego-Ortega *et al*. 2015). ELF5 is a well-known master regulator of mammary progenitor cell fate (Oakes *et al*. 2008*a*, Gallego-Ortega *et al*. 2013, Lee *et al*. 2013). Its expression is altered in multiple cancers (Piggin *et al*. 2016); in breast cancer, elevated ELF5 suppresses oestrogen sensitivity and correlates with the ER− negative basal subtype (Kalyuga *et al*. 2012). In luminal breast cancer, progesterone signalling induces Elf5 and activates a number of immune functions (Hilton *et al*. 2010). Furthermore, ELF5 drives the expression of a number of factors implicated in MDSC recruitment and activation, presumably by direct transcriptional activation (Gallego-Ortega *et al*. 2015). Interestingly, Elf5 promotes resistance to endocrine therapy through a mechanism that involves direct transcriptional repression of ER signalling (Kalyuga *et al*. 2012). It is tempting to speculate that these two aspects of aggressive disease, MDSC-driven immunosuppression and promotion of resistance to endocrine therapy are linked (Fig. 2) and that an ER-suppressive environment is especially conducive to MDSC recruitment. The stromal microenvironment of ER+ and ER− tumours is markedly different, including the higher expression of MDSC-recruiting CCL2 in ER− tumours (Bianchini *et al*. 2010).

The expansion and activation of the MDSC population is influenced by a complex network of signals and factors that are categorised into two groups. The first group involves factors that are secreted by tumour cells to induce the expansion of MDSCs and their accumulation in tumours. The second group of factors are produced by the tumour stroma and T-cells to activate MDSCs (Condamine *et al*. 2015).

Factors involved in the expansion of the MDSCs include GM-CSF, M-CSF and VEGF. The receptors for these factors on MDSC activate signalling to the nucleus via STAT3. STAT3 appears to be one of the primary transcription factors that regulate MDSC expansion (Fig. 2). In both *in vivo* mouse models and *in vitro* model in haematopoietic progenitor cells, STAT3 activation was associated with increased levels of MDSC. Inhibition of STAT3 signalling reduced the size of the MDSC population and allowed the elicitation of anti-tumour immunity (Nefedova *et al*. 2004, Kortylewski *et al*. 2005). As the target genes of STAT3 are associated with increased proliferation and pro-survival function, such as BCL-XL, survivin and cyclin D1, it is likely that STAT3 induces the expansion of MDSCs by promoting proliferation, while also blocking the differentiation of IMC into mature myeloid cells. Additionally, the pro-inflammatory proteins S100A8 and S100A9 are upregulated by STAT3 and have a role in the expansion of MDSC in tumours by preventing myeloid-cell differentiation and in abrogating T-cell function in breast cancer, ovarian cancer and gastric cancer (Arai *et al*. 2008, Sinha *et al*. 2008, Wang *et al*. 2013). A recent study by Kumar and coworkers suggested that the inhibition of STAT3 activity is also associated with the pathological differentiation of M-MDSCs into TAMs, which were the major population within tumours compared to M-MDSC (Kumar *et al*. 2016a) indicating a level of unforseen complexity in the regulation of tumour immunity. Other studies suggest that M-MDSCs and PMN-MDSCs may have distinct routes of pathological differentiation within the tumour microenvironment. Yu and coworkers demonstrated in tumour-bearing mice that the silencing of the retinoblastoma gene induced the differentiation of M-MDSC to acquire features that were morphologically and phenotypically similar to PMN-MDSC, but which had not acquired the functional activity of PMN-MDSC (Yu *et al*. 2013). Additionally, monocytes and M-MDSC that are recruited to tumour sites are suggested to differentiate into TAM, which allows their immunosuppressive functions to be exerted (Kumar *et al*. 2016*a*). Some studies suggest that the exposure of MDSC to the hypoxic microenvironment within the tumour allows hypoxia-inducible factor HIF1A to regulate and induce MDSC differentiation into TAM (Corzo *et al*. 2010, Liu *et al*. 2014). Interferon regulatory factor-8 (IRF8) is also a negative regulator of MDSC expansion, as IRF8 overexpression was found to attenuate MDSC accumulation in breast cancer and enhanced the responsiveness to immunotherapy (Waight *et al*. 2013).

### Immunosuppressive mechanisms of MDSCs

MDSCs have distinct biochemical and genetic features that provide their immunosuppressive effects and the inability to differentiate into mature myeloid cells. The activation of MDSCs is regulated by multiple signalling pathways that include STAT1, STAT6 and NFkB. Factors that initiate these pathways, including TLR, IL4, IL13, TGFB and IFNG, are expressed by tumour stroma and activated T-cells (Fig. 1). STAT1 is one of the major transcription factor that is facilitated by IFNG to upregulate the expression of arginase-1 (ARG1) and inducible nitric oxide synthase (iNOS) in MDSCs, which along with other factors, provide the basis of the immunosuppressive functions in MDSCs. Studies have previously described the mechanism by which MDSCs can induce anergy in both natural killer (NK) cells (Hoechst *et al*. 2009, Li *et al*. 2009) and in the adaptive immune system, in particular CD4+ and CD8+ T-cells (Nagaraj & Gabrilovich 2012). In the C26 colon adenocarcinoma mouse model, MDSCs have been suggested to lead to reduced IFN-driven responsiveness of T lymphocytes and NK cells (Mundy-Bosse *et al*. 2011). Within the tumour microenvironment, MDSCs can inhibit T-cell function and proliferation through several different mechanisms. MDSC highly expresses both ARG1 and iNOS, enzymes that are capable of metabolising l-arginine. iNOS converts l-arginine to nitric oxide (NO), and ARG1 converts l-arginine into urea and l-ornithine. l-arginine is an amino acid that is vital for T-cell activity, and deprivation of this amino acid in the microenvironment inhibits T-cell proliferation and function (Fletcher *et al*. 2015). Additionally, the production of NO through the metabolism of l-arginine by iNOS suppresses T-cell activity and also induces apoptosis (Bingisser *et al*. 1998, Rivoltini *et al*. 2002). MDSCs isolated from breast cancer tissue have Stat3-dependent upregulation of indole amine 2,3 dioxygenase (IDO), an enzyme responsible for the catabolism of tryptophan. The high expression of IDO depleted the tumour microenvironment of tryptophan and produced kynurenine-based byproducts, which led to the inhibition of T-cell proliferation and induced T-cell apoptosis. Furthermore, IDO induces CD4^+^ CD25^+^ T regulatory cell infiltration to primary breast tumours and lymph node metastases (Curti *et al*. 2007, Yu *et al*. 2013).

MDSCs also have upregulated activity of NADPH oxidase (NOX2), resulting in the increased generation of ROS in the form of superoxide anion (O_2_^−^) and peroxynitrite (ONOO^−^). The elevated production of ROS by MDSC creates oxidative stress within the tumour microenvironment and is a major component of the immunosuppressive effects of MDSCs on the antigen-specific response of T-cells and the inhibition of MDSC differentiation to mature myeloid cells (Kusmartsev & Gabrilovich 2003, Kusmartsev *et al*. 2004). Peroxynitrite, a powerful oxidant synthesised in the reaction between NO and superoxide anion, is another important mediator of the suppression of T-cell function and has been linked with T-cell deactivation in cancer. High levels of peroxynitrite are found in sites of expanded MDSC population and have been correlated with tumour progression and metastasis in cancer (Vickers *et al*. 1999, Nakamura *et al*. 2006). During direct cell-to-cell interaction between MDSCs and T-cells, peroxynitrite can chemically alter the T-cell receptor and CD8 molecule on the surface of T-cells by nitration. This modification renders the T-cell unresponsive to antigen-specific stimulation, but not to nonspecific stimuli (Nagaraj *et al*. 2007). This antigen-specific interaction is more stable and prolonged than nonspecific interactions, allowing the ROS and peroxynitrite to exert their effects on the surface of T-cells to suppress their antigen-specific response (Kusmartsev *et al*. 2000). In cancer patients, T-cells found in the peripheral blood are still capable of responding to other stimuli that are not tumour-specific antigens (Antonia *et al*. 2006, Mirza *et al*. 2006).

In most cancers, the PMN-MDSC population has been reported to outnumber the M-MDSC population (Gallego-Ortega *et al*. 2015, Messmer *et al*. 2015, Kumar *et al*. 2016*b*). Preferential expansion of a specific subtype of MDSC can be caused by factors that exist in the tumour microenvironment. For example, M-MDSCs are found as the predominant population in prostate cancer and PMN-MDSCs in breast cancer (Marvel & Gabrilovich 2015). However, the ratio of population of PMN-MDSCs to M-MDSCs is a vital factor in determining the different mechanisms that are utilised by the MDSC to suppress the immune response. PMN-MDSCs produce a higher amount of ROS in comparison to M-MDSCs, and as such, require cell-to-cell contact to exert suppression on antigen-specific response of T-cells. In contrast, M-MDSCs produce more ARG1, iNOS and immunosuppressive cytokines, such as TGFB, and suppresses the nonspecific T-cell response (Haverkamp *et al*. 2014, Kumar *et al*. 2016*b*). Additionally, the strength of immunosuppression in the MDSC subsets is primarily determined by GM-CSF secreted by tumours, and on a per-cell basis, M-MDSC possesses more potent suppressive activity compared to PMN-MDSC (Dolcetti *et al*. 2010, Kumar *et al*. 2016*b*). MDSC isolated from tumours were found to have stronger immunosuppressive activity compared to their peripheral counterparts (Qian *et al*. 2011, Haverkamp *et al*. 2014, Maenhout *et al*. 2014).

### Treatments targeting MDSC

Suppression of the immune system has been a major limitation for the successful treatment of cancer by immunotherapy. Indeed, as MDSCs are capable of subverting immunosurveillance and suppressing anti-tumour immunity, there has been more attention focusing on MDSC as a potential therapeutic target in pathological conditions, in particular, cancer. Clinical evidence shows a strong correlation between the tumour-induced MDSC dysfunction and poor patient prognosis (Messmer *et al*. 2015), including breast cancer (Diaz-Montero *et al*. 2009). Increased circulating MDSCs have been associated with decreased T-cell activation and with decreased efficacy of immunotherapeutic intervention (Gabrilovich & Nagaraj 2009, Ostrand-Rosenberg 2010, Gabrilovich *et al*. 2012). Elimination of MDSCs is effective in improving adoptive T-cell transfer therapy in breast cancer (Alizadeh & Larmonier 2014). Therapeutic targeting of MDSCs has a remarkable potential as a therapy for breast cancer, by either promoting MDSC differentiation to a non-suppressive phenotype or by eliminating MDSCs through activation of their apoptosis program or inhibition of their production from haematopoietic stem cells.

### Modulation of MDSC immunosuppressive functions

Promoting MDSC to differentiate into mature myeloid cells removes their suppressive functions. One such approach is using all-trans-retinoic acid (ATRA). ATRA is an agonist of nuclear retinoid receptors, such RARA, which are responsible for facilitating the differentiation of IMC into mature myeloid cells (DC and macrophages), thus removing their immunosuppressive activity. Administration of ATRA in mice or patients caused MDSC differentiation seen as increased expression of phenotypic markers associated with mature myeloid cells. In these cases, there was a lower MDSC population in peripheral blood and better antigen-specific immune response (Bastien & Rochette-Egly 2004, Hengesbach & Hoag 2004, Kusmartsev *et al*. 2008). The reduction in MDSC by ATRA improves tumour-specific response in T-cells (Mirza *et al*. 2006), and in tumour models, combination of ATRA with cancer vaccines significantly prolonged the anti-tumour effect of the treatment (Kusmartsev *et al*. 2003). This strategy is being tested in clinical trials. The mechanism of action for ATRA reduction of MDSC is not clear but may involve glutathione synthase (GSS) (Nefedova *et al*. 2007).

By blocking the signalling pathways that regulate the expression of ARG1 and iNOS, the immunosuppressive activity of MDSC can be inhibited. COX2 can promote the expression of ARG1 in MDSC, thus inducing their suppressive function. Celecoxib, a COX2 inhibitor, enhances efficacy of immunotherapy by repressing ARG1 expression and improving T-cell response to tumour-specific antigen (Talmadge *et al*. 2007). Recently, Zelenay and coworkers had demonstrated that both COX1 and COX2 can be partially inhibited with the addition of aspirin, and when mice that were implanted with melanoma were treated in combination of anti-PD-1 monoclonal antibody and aspirin, a marked reduction in tumour regression and eradication of melanoma cells was observed compared with just the anti-PD-1 alone (Zelenay *et al*. 2015). This synergistic effect was also observed when mice were administered with celecoxib and treated in conjunction with anti-PD-1, but to a lesser degree compared with aspirin (Zelenay *et al*. 2015).

Additionally, phosphodiesterase-5 (PDE-5) inhibitors and nitroaspirin limit the expression or activity of ARG1 and iNOS, leading to improved responsiveness and population of T-cells (Serafini *et al*. 2006, Wesolowski *et al*. 2013, Califano *et al*. 2015). Increased production of ROS and NO hampers T-cell responsiveness through nitration of the T-cell receptor. Anti-inflammatory triterpenoids, such as CDDO-IM and CDDO-Me, inhibits the immunosuppressive activity of MDSC by upregulating the transcription factor Nf-E2-related factor 2 (NRF2), which has been found to play a role in the protection of cells against oxidative stress. NRF2 regulates the expression of several antioxidant genes such as NADPH, NQO1 and hemeoxygenase and increased expression of NRF2 results in a reduction of intracellular ROS and attenuates MDSC-driven immunosuppression (Thimmulappa *et al*. 2007, Nagaraj *et al*. 2010, Marvel & Gabrilovich 2015), whereas deletion of NRF2 was reported to increase metastasis in mice with Lewis lung carcinoma due to the aberrant accumulation of ROS in MDSC (Hiramoto *et al*. 2014). Contradicting this, other studies have reported that permanent activation of NRF2, and subsequently enhanced ROS detoxification, in human lung carcinomas has also been found to promote tumourgenesis, pulmonary malignancy and resistance to chemotherapy (Singh *et al*. 2008, Bauer *et al*. 2011).

Different studies have shown both the beneficial and detrimental aspects of using antioxidants, such as N acetyl-cysteine (NAC) and vitamin E, as an anti-cancer treatment. NAC and vitamin E have been shown to reduce the immunosuppressive activity of MDSC by scavenging free radicals, decreasing MDSC population in the tumour microenvironment and increasing the population of activated T-cells both *in vivo* and *in vitro* (Srivastava *et al*. 2010, Kang *et al*. 2014). Conflicting studies have also suggested that the use of antioxidants may promote tumour growth and increase metastasis. Addition of NAC and vitamin E in the diet of mice with BRAF- and KRAS-induced lung cancer was shown by Sayin and coworkers to increase tumour cell proliferation by decreasing p53 expression, subsequently promoting tumour growth (Sayin *et al*. 2014). Additionally, administration of antioxidants in mice with malignant melanoma was reported to promote lymph node metastases but did not affect the growth of the primary tumours (Le Gal *et al*. 2015, Piskounova *et al*. 2015). In breast cancer, the effects of antioxidants have remained controversial regarding the risk of recurrence and mortality among premenopausal and postmenopausal women (Fleischauer *et al*. 2003, Cui *et al*. 2008, Pan *et al*. 2011).

### Apoptosis of MDSC

An increasing number of chemotherapeutic drugs activate tumour immune rejection by targeting MDSC, suggesting that part of their anti-tumour success includes reactivation of the immune system (Naiditch *et al*. 2011). Gemcitabine, has been utilised in tumour-bearing mice to specifically lower the population of MDSC in the spleen, and was effective in reducing tumour growth and increasing anti-tumour immune activity (Suzuki *et al*. 2005,^ 2007^, Le *et al*. 2009). Cisplatin and 5-fluorouracil have also been used to successfully deplete MDSCs and improve T-cell responsiveness (Tseng *et al*. 2008, Vincent *et al*. 2010). Doxorubicin promoted apoptosis of MDSCs and interfered with the suppressive ability of MDSCs and restored T-CD8+ lymphocyte responses (Alizadeh *et al*. 2014). Docetaxel administration significantly inhibited tumour growth in 4T1 tumour-bearing mice and decreased the numbers of MDSCs in the spleen. The treatment also selectively increased CTL responses and polarised MDSC towards an anti-tumourigenic phenotype (Kodumudi *et al*. 2010). Interestingly, epigenetic modulators such as 5-azacytidine and 5-aza-2′-deoxy-azacytidine have also resulted in MDSCs killing (Kim *et al*. 2014).

The opposite effect of chemotherapy on MDSCs has also been demonstrated. For example, although cyclophosphamide has been proposed to enhance cancer vaccines presumably by its effect on Tregs (Machiels *et al*. 2001, Lutsiak *et al*. 2005), in non-tumour-bearing animals, it leads to transient surges in MDSC (Angulo *et al*. 2000, Salem *et al*. 2007). Breast cancer patients receiving cyclophosphamide as part of their chemotherapy had a five-fold increase in circulating MDSCs in blood, and this increase was associated with low T-cell activity (Diaz-Montero *et al*. 2009). This indicates that immune modulation is a double-edged sword and that methods to characterise the immune landscape of the patient would be very informative before the administration of these drugs.

## Concluding remarks

Two interconnected layers of immune populations operate in cancer, the innate and the adaptive immune system. Immunotherapies aimed at reactivating the tumour-rejecting cytotoxic capacity of T-cells are efficient in types of cancer with a high mutational profile. Breast tumours have relatively low TIL infiltration, consequently T-cell-directed therapies, such as checkpoint inhibitors, have not resulted in major responses. The components of the innate immune system have a prominent role during breast cancer progression, and this might reflect the importance of the innate immune system in normal mammary gland development that couples tissue morphogenesis with immunosuppression. During mammary involution, neutrophils (the precursors of MDSC) are recruited but maintained in an immunosuppressive environment. It is possible that the same mechanisms are hijacked by breast cancer cells to increase tumour tolerance and promote T CD8+ cell exclusion. MDSCs have been shown to regulate T-cell exclusion by a variety of mechanisms, thus representing promising targets for therapy. Immunotherapies directed to the intersection of the two layers of the immune system may open new avenues for the treatment of breast cancer patients. MDSC is a compelling target for cancer therapy, but their heterogeneous nature and poor definition in humans make this elusive. A transcriptional definition of tumour-infiltrating MDSCs may better characterise and classify this heterogeneous population, laying the foundation to specifically target pro-tumorigenic MDSC populations and unleashing the development of breast cancer immunotherapies.

## Declaration of interest

The authors declare that there is no conflict of interest that could be perceived as prejudicing the impartiality of this review.

## Funding

This work has been financially supported by the National Health and Medical Research Australia, grant number APP1068753. DGO is supported by a Garvan Foundation Fellowship generously sponsored by May Dariymple. CJO is supported by the National Health and Medical Research Australia Fellowship APP1043400. EL is supported by the National Breast Cancer Foundation/Victorian Cancer Agency PF14-002.
