# The innate and adaptive infiltrating immune systems as targets for breast cancer immunotherapy

**DOI:** 10.1530/ERC-16-0404e

**Published:** 2017-07-01

**Authors:** Andrew M K Law, Elgene Lim, Christopher J Ormandy, David Gallego-Ortega

**Affiliations:** 1 Tumour Development GroupThe Kinghorn Cancer Centre, Garvan Institute of Medical Research, Darlinghurst, New South Wales, Australia; 2 Cancer Biology LaboratoryThe Kinghorn Cancer Centre, Garvan Institute of Medical Research, Darlinghurst, New South Wales, Australia; 3 Connie Johnson Breast Cancer Research LaboratoryThe Kinghorn Cancer Centre, Garvan Institute of Medical Research, Darlinghurst, New South Wales, Australia; 4 St. Vincent’s Clinical SchoolFaculty of Medicine, University of New South Wales Australia, Sydney, New South Wales, Australia

The authors and journal apologise for an error in the above paper, which appeared in volume 24 part 4, pages R123–R144. The error relates to the Funding section on page R135.

The original paper stated:

DGO is supported by a Garvan Foundation Fellowship generously sponsored by May Dariymple.’

This should have stated:

DGO is supported by the Garvan Research Foundation and generous donor, Mrs May Dalrymple.’

